# Clinical discussion of the arteria lusoria: a case report

**DOI:** 10.1590/1677-5449.007617

**Published:** 2017

**Authors:** Tulio Fabiano de Oliveira Leite, Lucas Alves Sarmento Pires, Rafael Cisne, Marcio Antonio Babinski, Carlos Alberto Araujo Chagas

**Affiliations:** 1 Universidade de São Paulo – USP, Faculty of Medicina, Interventional Radiology, São Paulo, SP, Brazil.; 2 Universidade Federal Fluminense – UFF, Medical Sciences Post Graduation Program, Niterói, RJ, Brazil.; 3 Universidade Federal Fluminense – UFF, Morphology Department, Niterói, RJ, Brazil.

**Keywords:** arteria lusoria, right subclavian artery, anatomical variation, case report, artéria lusória, artéria subclávia direita, variação anatômica, relato de caso

## Abstract

The right subclavian artery may originate from the left portion of the aortic arch. This aberrant vessel is known as the arteria lusoria. Its course to its usual site runs behind the esophagus, which may cause a disease known as dysphagia lusoria, responsible for symptoms of discomfort. This artery is often associated with other anomalies, such as the non-recurrent laryngeal nerve and the bicarotid trunk, and with diseases such as aneurysms, congenital heart defects, and even genetic syndromes. During routine dissection of a male cadaver fixed in 10% formalin solution, an arteria lusoria was found. This article reports the variation and discusses its embryological, clinical and surgical aspects.

## Resumo

Em alguns casos, a artéria subclávia direita pode se originar da porção esquerda do arco aórtico. Esse vaso aberrante é conhecido como artéria lusória. Para chegar em sua região, essa artéria passa posteriormente ao esôfago, e pode ser, portanto, causa de uma doença conhecida como disfagia lusória, desencadeando sintomas desconfortantes. A artéria lusória está frequentemente associada com outras anomalias, como o nervo laríngeo não recorrente e o tronco bicarotídeo, assim como pode estar em associação com aneurismas, defeitos cardíacos congênitos e até síndromes genéticas. Durante dissecação cadavérica de rotina, foi observada a presença dessa artéria em um cadáver do sexo masculino fixado em uma solução de formalina a 10%. O objetivo deste trabalho é relatar a variação conhecida como artéria lusória e trazer destaque para seus aspectos embriológicos, clínicos e cirúrgicos.

## INTRODUCTION

The aortic arch (AA) usually gives off three branches: the brachiocephalic trunk (from which originate the right subclavian artery [RSA] and right common carotid artery), the left common carotid artery, and, lastly, the left subclavian artery, from right to left.^1^ Variations of the AA and its branches are well known in the literature.^1^^,^ 2

The aberrant right subclavian artery (ARSA) or right subclavian retroesophageal artery (RSRA) occurs in 0.5% to 2.5% of cases. It is known as the “arteria lusoria” (AL).^3^

This vessel’s route to its usual site usually takes a retroesophageal path, although it can sometimes pass anteriorly to the trachea or even between these two structures.^1^^,^ 3

Due to this trajectory, the AL is of clinical interest because it can cause esophageal compression and symptoms of dysphagia - a condition known as dysphagia lusoria - or dyspnea.^3^^-^ 6

This aberrant vessel also has surgical significance, because of its spatial relations to many structures, and it can be damaged during surgical procedures.^7^^,^ 8

Although it is usually silent and most cases observed are incidental findings during autopsies, the variation is commonly seen together with a non-recurrent laryngeal nerve and other embryological development abnormalities of the AA complex or the carotid or pulmonary systems.^9^^,^ 10

Studies show that the arteries derived from the fourth arch may have abnormalities in their walls, which is why they could be subject to specific anomalies and pathologies and aortic tears or dissection are also more likely to occur when the AL is present.^11^^,^ 12 Additionally, Kommerel’s diverticulum may also coexist with the AL.^12^

This article reports an AL found in a male cadaver and discusses its embryological, clinical, and surgical aspects.

## CASE REPORT

A cadaver fixed in 10% formalin solution was dissected during a regular anatomy class. After routine dissection of the right upper limb, the axillary artery was traced and the right subclavian artery was dissected. It was observed that the artery did not originate from the brachiocephalic trunk as usual.

**Figure 1:**
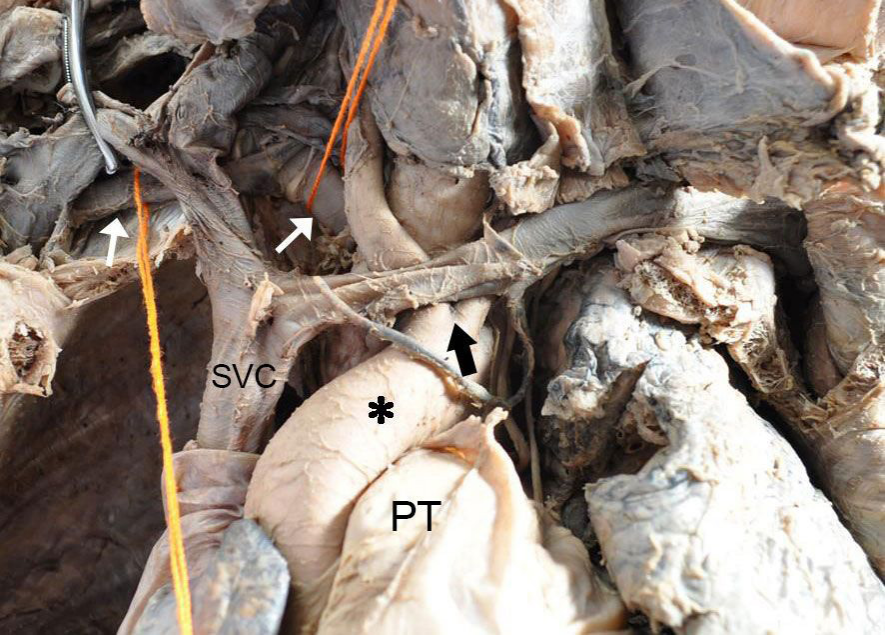
Anterior view of the arteria lusoria. Black asterisk, aortic arch; black arrow, bicarotid trunk; white arrows, arteria lusoria; PT: pulmonary trunk; SVC: superior vena cava.

Further dissection revealed that the vessel arose from the distal portion of the AA and followed a retroesophageal trajectory, while the AA presented a bicarotid trunk. The non-recurrent laryngeal nerve was present (Figure 1). No other variations were observed.

## DISCUSSION

There are six aortic arches during embryonic development. Some of those arches are lost at different stages of embryogenesis. The fourth AA gives origin to the regular AA of the fetus. The RSA arises from the fourth arch, the right dorsal aorta and the right seventh intersegmental artery.^13^^,^ 14

Essentially, the AL is a remnant of the right dorsal aorta caudal to the seventh intersegmental artery. This embryonic alteration is associated with disappearance of the normally patent right fourth AA and part of the right dorsal aorta cranial to the seventh intersegmental artery during embryogenesis.^15^

The AL was firstly described by Hunauld in 1735, although David Bayford described the first case of dysphagia lusoria in 1794. The word lusoria is derived from the Latin expression “*lusus naturae*,” which means “trick of nature”.^3^

According to the literature, incidence of the AL ranges from 0.2% to 3%.^6^^,^ 12 ^,^ 15 ^-^ 17 The trajectory of the AL can be retroesophageal (80-84%), pretracheal (4.2-5%), or passing between the two structures (12.7-15%).^3^^,^ 12

Regarding its origin, it usually originates from the upper portion of the thoracic aorta or as the leftmost branch of the AA.^1^^,^ 12 Moreover, it can be dilated at its origin, forming a diverticulum known as “Kommerel’s Diverticulum,” first described by Burckhard F. Kommerell in 1936.^3^ The incidence of this diverticulum was reported as 60% by Myers et al.,^3^ although Polguj et al.^12^ reports an incidence of 14.9% in a review of 141 cases.

**Figure 2:**
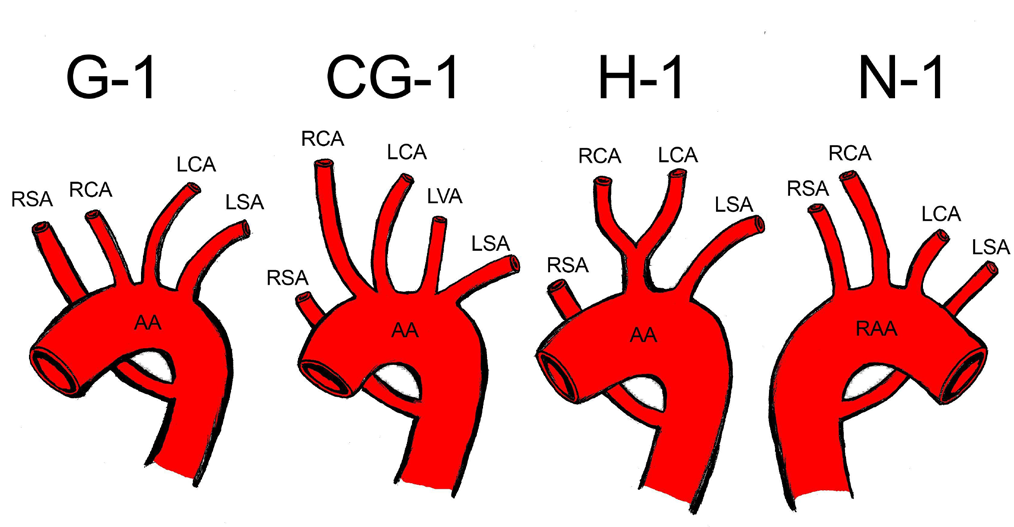
Schematic drawing based on the Adachi-Williams classification of right subclavian artery anomalies. AA: aortic arch; RAA: right aortic arch; RSA: right subclavian artery; RCA: right common carotid artery; LCA: left common carotid artery; LVA: left vertebral artery; LSA: left subclavian artery.

Adachi and Williams classified the numerous variations of the AA branching pattern. They also classified the RSRA into four different types: 1) Type G-1, in which the ARSA arises from the distal portion of the AA as its last branch. The other main branches have no variations; 2) Type CG-1, in which the ARSA originates from the distal portion of the AA and the left vertebral artery originates directly from the AA; 3) Type H-1, in which the AL also arises from the distal portion of the AA, although a bicarotid trunk is also present (as observed in this case); 4) Type N-1, in which there is a mirror image of type G, with a right-sided AA and the left subclavian artery mimicking the AL.^10^^,^ 18 Those types are illustrated in Figure 2.

A systematic review by Polguj et al.^12^ reports a higher incidence of the AL in females (55.3%) than in males (44.7%). The AL is usually asymptomatic.^3^^,^ 12 The review showed that the most common symptoms are dysphagia (71.2%), dyspnea (18.7%), retrosternal pain (17.0%), coughing (7.6%), and weight loss (5.9%), although symptoms such as stomach-ache, back pain, and numbness of the right upper limb were also described.^12^ Clinically, AL may mimic pericarditis, endocarditis, or aortic dissection.^19^

AL is usually seen together with other anatomical variations, such as bicarotid trunk, non-recurrent laryngeal nerve, and right-sided AA. Furthermore, it can be associated with many cardiac anomalies (aortic coarctation, interrupted AA, tetralogy of Fallot, truncus arteriosus, transposition of the great arteries, and ventricular and atrial septal defects), genetic disorders such as Downs’, Edwards’, and DiGeorge syndromes, aneurysms, and arterioesophageal fistula.^3^^,^ 8 ^,^ 9 ^,^ 12 ^,^ 20 ^,^ 21

This vessel is of considerable surgical interest because of its spatial disposition, since it can be injured during many head and neck surgeries, such as lymph node dissection of the right paratracheal fossa or thyroidectomies, tracheotomy, and transradial coronary procedures.^7^^-^ 9 The AL can be used as a flap in order to treat aortic coarctation.^22^

Dysphagia lusoria is usually treated with endovascular techniques, thoracic endografts and revascularization, ligation of the AL via left thoracotomy, and even embolization.^12^^,^ 16

## CONCLUSION

The AL is a clinically significant anatomical variation, since it can mimic many different diseases, as well as cause dysphagia in patients. Furthermore, presence of the AL seems to predispose to aneurysms. Knowledge of this variation is of crucial importance to radiologists, head and neck surgeons, vascular surgeons, and clinicians.
